# Impact of Hydroxychloroquine on Atherosclerosis and Vascular Stiffness in the Presence of Chronic Kidney Disease

**DOI:** 10.1371/journal.pone.0139226

**Published:** 2015-09-28

**Authors:** Ashutosh M. Shukla, Chhanda Bose, Oleg K. Karaduta, Eugene O. Apostolov, Gur P. Kaushal, Tariq Fahmi, Mark S. Segal, Sudhir V. Shah

**Affiliations:** 1 North Florida/South Georgia Veterans Healthcare System, Gainesville, Florida, United States of America; 2 University of Florida, Gainesville, Florida, United States of America; 3 Central Arkansas Veterans Healthcare System, Little Rock, Arkansas, United States of America; 4 University of Arkansas for Medical Sciences, Little Rock, Arkansas, United States of America; University of Louisville, UNITED STATES

## Abstract

Cardiovascular disease is the largest cause of morbidity and mortality among patients with chronic kidney disease (CKD) and end-stage kidney disease, with nearly half of all deaths attributed to cardiovascular disease. Hydroxychloroquine (HCQ), an anti-inflammatory drug, has been shown to have multiple pleiotropic actions relevant to atherosclerosis. We conducted a proof-of-efficacy study to evaluate the effects of hydroxychloroquine in an animal model of atherosclerosis in ApoE knockout mice with and without chronic kidney disease. Forty male, 6-week-old mice were divided into four groups in a 2 x 2 design: sham placebo group; sham treatment group; CKD placebo group; and CKD treatment group. CKD was induced by a two-step surgical procedure. All mice received a high-fat diet through the study duration and were sacrificed after 16 weeks of therapy. Mice were monitored with ante-mortem ultrasonic echography (AUE) for atherosclerosis and vascular stiffness and with post-mortem histology studies for atherosclerosis. Therapy with HCQ significantly reduced the severity of atherosclerosis in CKD mice and sham treated mice. HCQ reduced the area of aortic atherosclerosis on *en face* examination by approximately 60% in HCQ treated groups compared to the non-treated groups. Additionally, therapy with HCQ resulted in significant reduction in vascular endothelial dysfunction with improvement in vascular elasticity and flow patterns and better-preserved vascular wall thickness across multiple vascular beds. More importantly, we found that presence of CKD had no mitigating effect on HCQ’s anti-atherosclerotic and vasculoprotective effects. These beneficial effects were not due to any significant effect of HCQ on inflammation, renal function, or lipid profile at the end of 16 weeks of therapy. This study, which demonstrates structural and functional protection against atherosclerosis by HCQ, provides a rationale to evaluate its use in CKD patients. Further studies are needed to define the exact mechanisms through which HCQ confers these benefits.

## Introduction

Cardiovascular disease (CVD) is the largest cause of morbidity and mortality[1, 2] among patients with chronic kidney disease (CKD) and end-stage kidney disease (ESKD), with nearly half of all deaths attributed to CVD[1]. Though no single factor thus far has been identified as the driver for this cardiovascular (CV) burden; inflammation, vascular stiffness likely resulting from endothelial dysfunction, and accelerated atherosclerosis are considered prominent factors contributing to the high rates of CVD in CKD.

Therapeutic interventions conventionally proven to be effective in CVD among patients with normal renal functions, have limited if any efficacy in presence of significant CKD. For example, HMG coenzyme A inhibitors, statins, when studied in CKD and ESKD populations have failed to show a significant reduction in CV mortality[3–7], even though some data suggest that they might be effective in reducing the rates for CV events[4]. When studied in animal models of atherosclerosis, statins failed to reduce the severity of atherosclerosis in presence of uremia; a sharp departure from their proven efficacy in presence of normal renal function[8]. Similarly, many therapies targeted toward management of renal failure (such as quotidian dialysis)[9], or its consequences (bone-mineral disease, anemia, etc.) have also failed to reduce the high CV mortality associated with CKD[3, 4, 7, 9, 10].

These factors have led to a high CV mortality in CKD[2] that has remained largely unchanged over the last two decades,[11] and at present time we do not have an effective management strategy that reduces the high CV mortality in CKD and ESKD. Thus there is an urgent need to investigate unique therapies that are efficacious against atherosclerosis and vascular disease, not just in presence of normal renal function, but also in presence of uremic milieu. Most patients suffering from CKD have multiple traditional and nontraditional CV risk factors such as chronic inflammation, oxidative stress, endothelial dysfunction, reduced vascular compliance, insulin resistance, and metabolic syndrome[12]and these factors have been repeatedly shown to contribute not only toward the pathogenesis of CVD in CKD but also a state of refractoriness toward conventional therapies[13].

Hydroxychloroquine (HCQ) is an anti-malarial drug that is commonly used in clinical practice for its anti-inflammatory actions. Multiple *in vivo*, *in vitro*, and cohort-based reports over the last two decades show that, in addition to being anti-inflammatory, HCQ has multiple other properties that include beneficial effects on vascular compliance and endothelial function,[14] insulin resistance, metabolic syndrome,[15–17] and immune dysfunction.[18, 19] Recently when studying the p-53 based stress signaling in the metabolic syndrome, investigators have found signals for a possible anti-atherosclerosis effect for HCQ in presence of normal renal function[17]. However, as the statin experience suggests, the efficacy in presence of normal renal function does not automatically prove the efficacy in presence of uremic milieu and the impact of HCQ as an anti-atherosclerosis as well as vasculoprotective agent in a uremic milieu has not been examined previously. We present the findings of our proof-of-efficacy animal study in ApoE-/- mice aimed to examine whether HCQ has beneficial effects on atherosclerosis and vascular disease in presence of a uremic state.

## Materials and Methods

Mice were maintained in the Veterans Administration Medical Unit (VAMU) at Central Arkansas Veterans Healthcare System, Little Rock, Arkansas, in accordance with the U.S. National Institutes of Health Guide for the Care and Use of Laboratory Animals and the protocol was approved by the Institutional Animal Care and Use Committee (IACUC #12-11-1) of Central Arkansas Veteran Healthcare System on animal care. Forty-two male, 6- to 8-week-old ApoE-/- mice with a C57BL6 background (Jackson Laboratories, Bar Harbor, ME) were divided into two different groups after an initial week of acclimatization: a CKD group (n = 26) and a sham group (n = 16). Mice in the CKD group underwent a two-step surgical process (cortical electrocoagulation of 80% of the right kidney followed two weeks later by a left kidney total nephrectomy), originally described by Gagnon et al.[20] and validated in our laboratories[21], to establish a reduced renal mass and induce CKD. The sham-operated mice underwent a right-flank incision and closure in the first stage of surgery and a left-flank incision and closure in the second stage of surgery and served as groups with normal renal function. All surgeries were performed under ketamine/xylazine (91.0/9.1 mg/kg, i.p.) anesthesia and all efforts were made to minimize the pain and potential distress by two post-operative analgesic injections of buprenorphine (0.05mg/kg, 0.1 ml) administered subcutaneously for one day.

All mice underwent serial blood work obtained by retro-orbital puncture for blood urea nitrogen and creatinine assessment every four weeks during the study period.

After surgery, all mice were fed a high-fat diet (Harlan Teklad, Madison, WI) with 21% total fat and 0.15% cholesterol to exacerbate hyperlipidemia and atherosclerosis development. Both CKD and sham groups of mice were divided further into those receiving HCQ (treatment groups) and those treated with placebo (controls), creating four final groups: (1) a sham control group (n = 8); (2) a sham treatment group (n = 8); (3) a CKD control group (n = 13); and (4) a CKD treatment group (n = 11). HCQ was administered in drinking water to achieve a therapeutic dose of 10 mg/kg/day. The dose of HCQ was selected based on the conventional low dose (6–10mg/kg/day) of HCQ used in human populations where the pleiotropic cardioprotective actions of HCQ have been demonstrated[14, 15, 22], and also in a prior animal study in a similar model in the presence of normal renal function[17]. The feasibility of HCQ mixed in the water supply and its effects on daily water intake were tested through a prior pilot observation study in the same strain of mice. Animals were group-housed in an animal care facility with 3–4 animals per cage.

Ante-mortem ultrasound echography (AUE) is an excellent tool to visualize the development and progression of atherosclerosis in mice and to evaluate the impact of modulating factors or interventions[23]. We performed an AUE of the aorta and major vessels at baseline, 12 weeks, and 16 weeks (just prior to sacrifice) into the study using the VisualSonics (Toronto, CAN) Vevo 770 in B mode (for the evaluation of the extent of atherosclerotic lesions), in M mode (for wall density and thickness at the pre-specified locations), and in Doppler mode (for rigidity and flow evaluations)[21, 24]. The detailed procedures and technique for this AUE examination have been published previously by our laboratory[21]. All mice were studied for a total duration of 16 weeks, with the last assessment for biochemical parameters and AUE at the time of sacrifice. Vascular wall thickness was obtained by the measurement of arterial wall thickness in 3–5 images taken of the vessel. Measurements and analysis were made separately for the upper and lower walls visible on AUE by an operator blinded to the identity of the animal.

Mice were euthanized at the end of sixteen weeks of therapy (at 23–24 weeks of age) with 300 mg/kg ketamine and 30 mg/kg 6 mg/kg xylazine (IP) followed by exsanguinations, and the sera and tissue were collected for the biochemical and histological assessments. Postmortem assessments included *en face* staining of the entire aortas with Sudan IV stain to detect the extent of atherosclerotic lesions (% of total aortic area with atherosclerotic lesions). Images of *en face* preparations of the whole aortas with atherosclerotic lesions were captured with a Nikon J1camera (Nikon, Melville, NY). The quantification of atherosclerotic lesions in full-length aorta, aortic arch, innominate artery (from the branching point to the Y-shaped bifurcation), thoracic aorta, and abdominal aorta were compared by using computerized analysis using the Image J. v 1.46 software (National Institutes of Health, Bethesda, MD), with lesions expressed as percentage of total vascular surface for each animal and the result was expressed as the percent of surface area of the entire aorta. For Oil Red O (Sigma, St. Louis, MO) staining for the cross-sectional analysis of lipid and fatty deposits in the aortic roots, the aorta along with the 3-mm lengths of ascending aorta (2 to 3 mm proximal to the brachiocephalic artery) was removed and frozen in OCT compound (Tissue-Tek, Sakura Finetek, Tokyo, Japan). 5 μm thick cryosections were stained with Oil Red O and counterstained with hematoxylin. For quantitative analysis of atherosclerosis, the total lesion area of the cross-sections was captured with a Nikon DS camera and analyzed by Image J. v 1.46 software. Mean plaque area of the aortic sinus was assessed and compared among the groups. Aortic roots (3 mm pieces of ascending aorta) were separated prior to *en face* staining and used for cryosectioning to assess atherosclerotic lesions in the aortic bulb. Additionally, mice sera were tested for lipids, IL-6 (Rey Biotech, Inc. Norcross, GA, cat. # ELM-IL-6-001), high sensitivity C-reactive protein (hsCRP; Antibodies-online, Inc. Atlanta, GA, cat. # ABIN415420), and soluble VCAM-1 (Quantikine ELISA kit, R&D Systems Inc., Minneapolis, MN cat. #MVC00) according to the manufacturers’ instructions.

### Statistical Analysis

All analyses were performed with the use of software GraphPad InStat 3, version 6.0 (GraphPad Software Inc., San Diego, CA) and SAS 9.4 (SAS Institute, Inc., Cary, NC). Continuous variables are expressed as mean ± SD and compared by Student’s t-test if it is independent and normally distributed, or Kruskal-Wallis test if it is independent but not normally distributed. The Friedman test was used to compare the significance of differences at multiple time points for elasticity and velocity. A two-way analysis of variance (ANOVA) was used to examine the main effects and the interactions of CKD and drug intervention on atherosclerosis and vascular thickness. Post-hoc Tukey’s and Dunn tests were applied when comparing three or more groups. A *P* value of < 0.05 was considered statistically significant.

## Results

Table 1 shows the morphologic and biochemical parameters among all four groups at the end of the 16 weeks of therapy. Two mice died due to anesthesia complications, leaving 8 mice each in both sham groups and eleven and thirteen mice in CKD treatment and control groups, respectively. There was no significant difference among the various groups for weight, daily water intake, and daily food intake. The water intake between all groups was similar and the final HCQ dosing was achieved as desired.

**Table 1:** Quantification of serum urea nitrogen (BUN), total bilirubin (TBIL), glucose (GLU) among all groups of mice at the time of sacrifice after 16 weeks of therapy with either HCQ or placebo.

	Sham control (n = 8)	Sham treatment (n = 8)	CKD control (n = 13)	CKD treatment (n = 13)
BUN (mg/dl)	20±1.4	26±2.0	90±12.3*	114±18.0^†^
TBIL (mg/dl)	0.33±0.0	0.33±0.0	0.35±0.0	0.4±0.0
GLU (mg/dl)	152±6.5	159±13.2	163±10.4	175±6.5
Body weight (g)	28.7 ±0.5	29±0.7	25.6±0.7	25.3±0.5
Food intake (g/24h)	3.3±0.5	3.0±0.7	2.9±0.4	3.0±0.6
Water/HCQ intake (ml/24h)	7.1±1.8	8.4±1	7.7±1.7	8.4±1.5

Body weight and daily food and water intake did not differ among different groups based on either presence or absence of CKD or therapy with HCQ or placebo.

Data presented as means ± SD

*P<0.001 versus sham control

†*P*<0.01 versus sham treatment by unpaired t-test.

### Impact of HCQ on atherosclerosis: antemortem and postmortem evaluations

**Fig 1:**
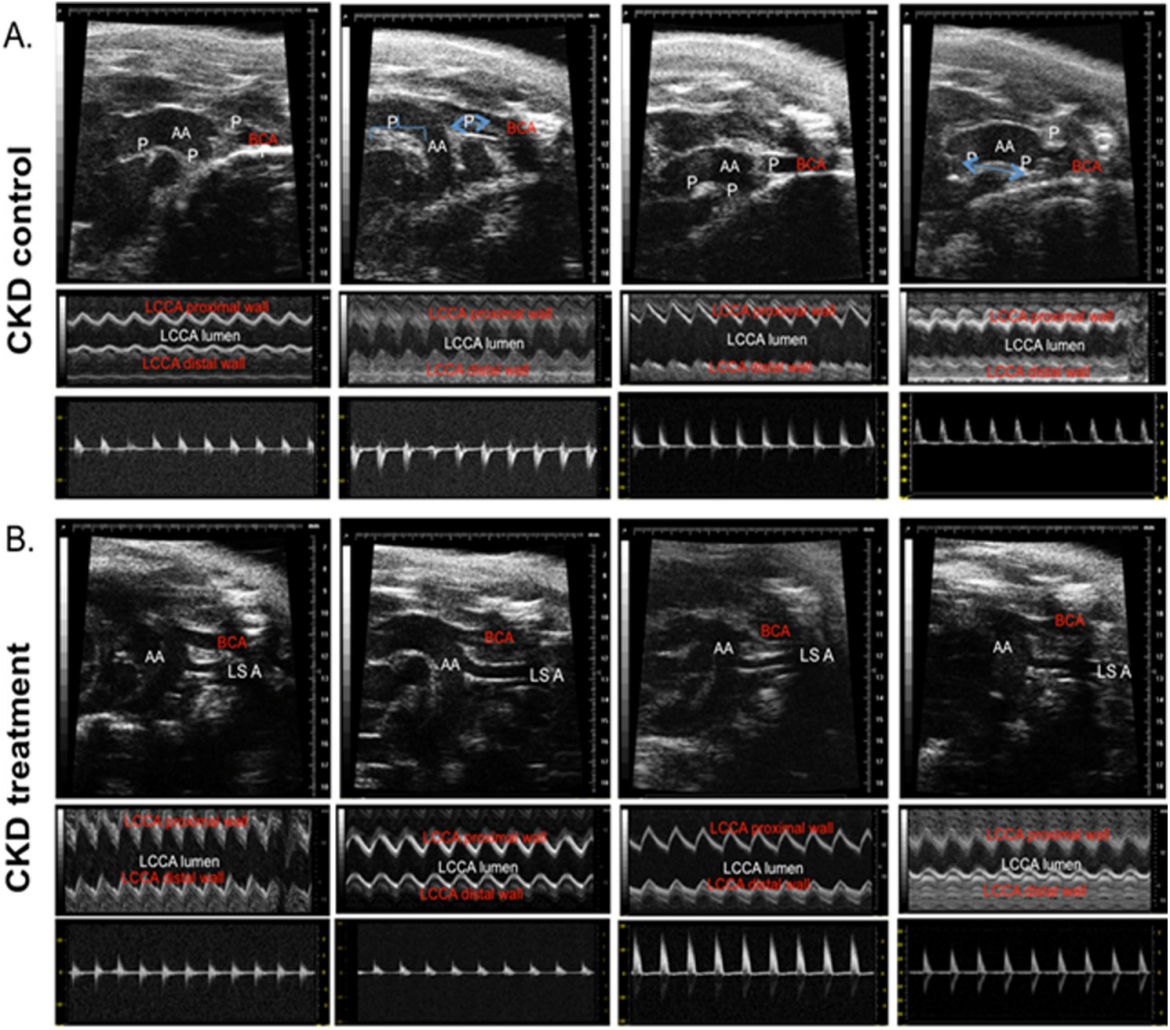
Antemortem echography (AUE; B, M, and Doppler mode) in CKD groups of mice at end of study. Representative images of aortic arch/ascending aorta and brachiocephalic arteries obtained by ante-mortem ultrasound echography (AUE) of CKD control (A) and CKD treated (B) mice, using VisualSonics Vevo 770. All mice were fed a high-fat diet and treated with 10 g/kg/day of HCQ for 16 weeks after CKD surgery. Data is shown for four control and four treated mice (n = 13, CKD control; n = 11, CKD treatment) groups. Blood velocity, arterial and aortic wall elasticity measurements in aortic arches/ascending aortas brachiocephalic arteries, left carotid arteries, left subclavian arteries and abdominal aortas were visualized by B-mode, Doppler mode, and M-mode. P, atherosclerotic plaque; AA, aortic arch; BCA, brachiocephalic artery; LCAA, left common carotid artery; LSA, left subclavian artery.

Fig 1 shows the ante-mortem AUE evaluation for aorta and major vessels in B and M mode echography and Doppler of the major vessels in the CKD groups of mice at the end of 16 weeks of therapy. The AUE studies were performed at baseline and 12 and 16 weeks into therapy just prior to sacrifice. The area of atherosclerosis was measured by identification of echo-dense atherosclerotic plaques on B-mode echography of the great vessels. We found that the extent of atherosclerosis on AUE examination was significantly reduced in mice receiving HCQ therapy (Fig 1B) compared to the controls (Fig 1A) at 12 weeks into the therapy. This continued to be evident on the examination performed at the end of the study at 16 weeks of therapy. The beneficial effects were evident in mice with CKD and with normal renal function.

**Fig 2:**
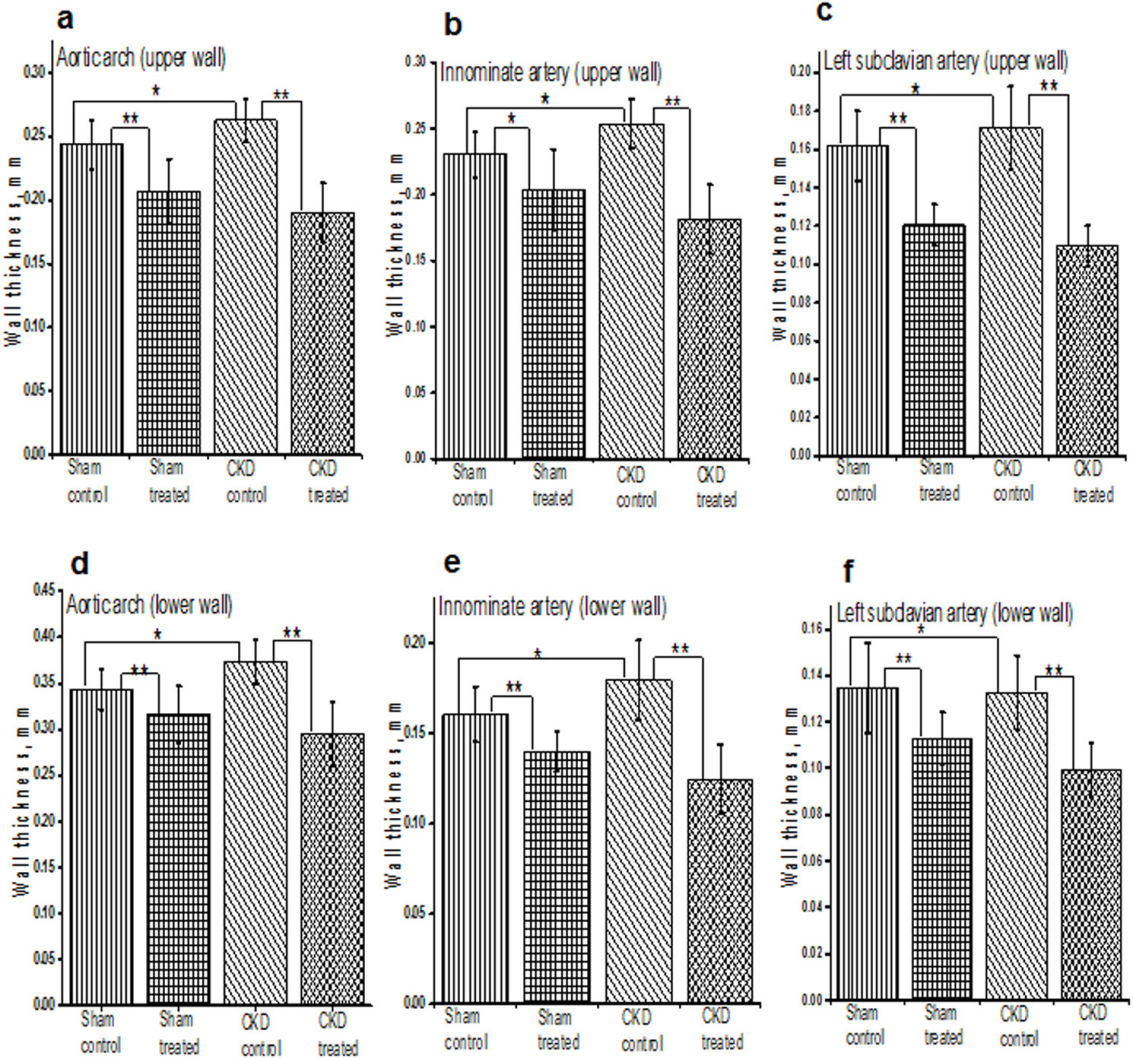
Histograms shows the quantitative analysis of mean arterial wall thickness in treated and untreated groups of sham and CKD mice. **a-f.** Data presented here are mean ± SD. (n = 8 for sham placebo and sham HCQ, n = 13 CKD placebo, and n = 11 CKD HCQ groups), *p<0.01 **p<0.001 as compared to the control group. For normally distributed data, unpaired 1-way analysis of variance (ANOVA) with Tukey-Kramer multiple comparisons test was used to test wall thicknesses in the 4 different groups of mice when significant (*P*<0.05). Post hoc analysis with unpaired Student *t* tests were used when comparing control and treated group.

We also measured the vascular wall thickness at pre-specified locations at their origin through the M-mode echo examination on AUE at the aortic arch and branching of major vessels (e.g. common carotid artery, left subclavian artery, and innominate artery). The progression of atherosclerosis was associated with progressive thickening of the vascular wall in all groups of mice. The vessel wall thickness was significantly lower among mice treated with HCQ compared to those that were not treated with HCQ (Fig 2), both in mice with normal renal function as well as those with CKD. The results from two-way ANOVA analyses further confirmed that HCQ therapy significantly associated with better preservation of thickness on these vascular walls (*P* < .0001 for aortic arch, left subclavian artery, and innominate artery).

**Fig 3:**
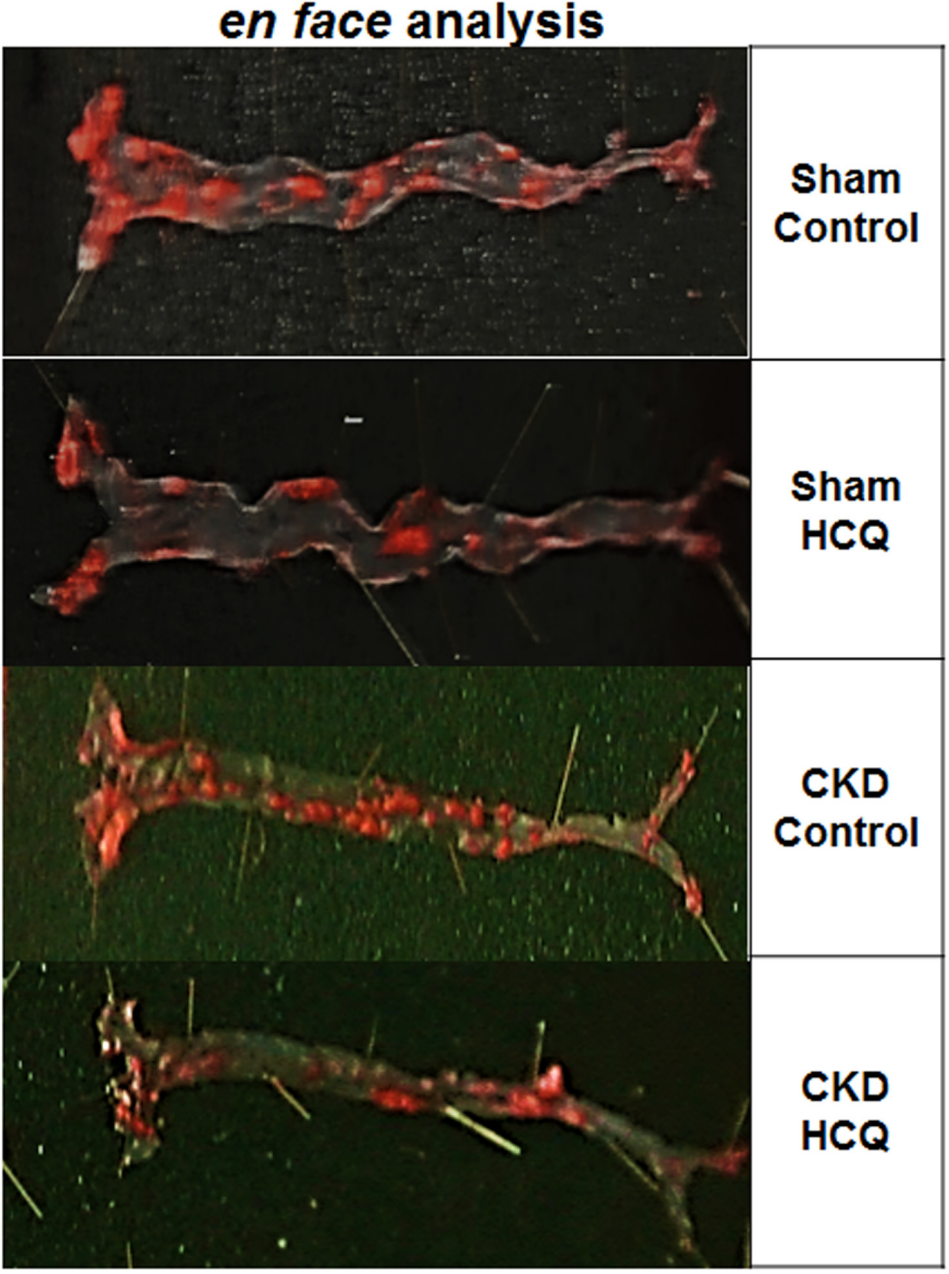
*En face* analysis of dissected entire aortic specimens in all groups of mice at the end of 16 weeks of therapy. Representative images of the Sudan IV staining for *en face* analysis of the total aorta. Atherosclerotic plaques are stained in orange/red. (n = 8 sham placebo and sham HCQ, and n = 13 CKD placebo, n = 11 CKD treated groups).

**Fig 4:**
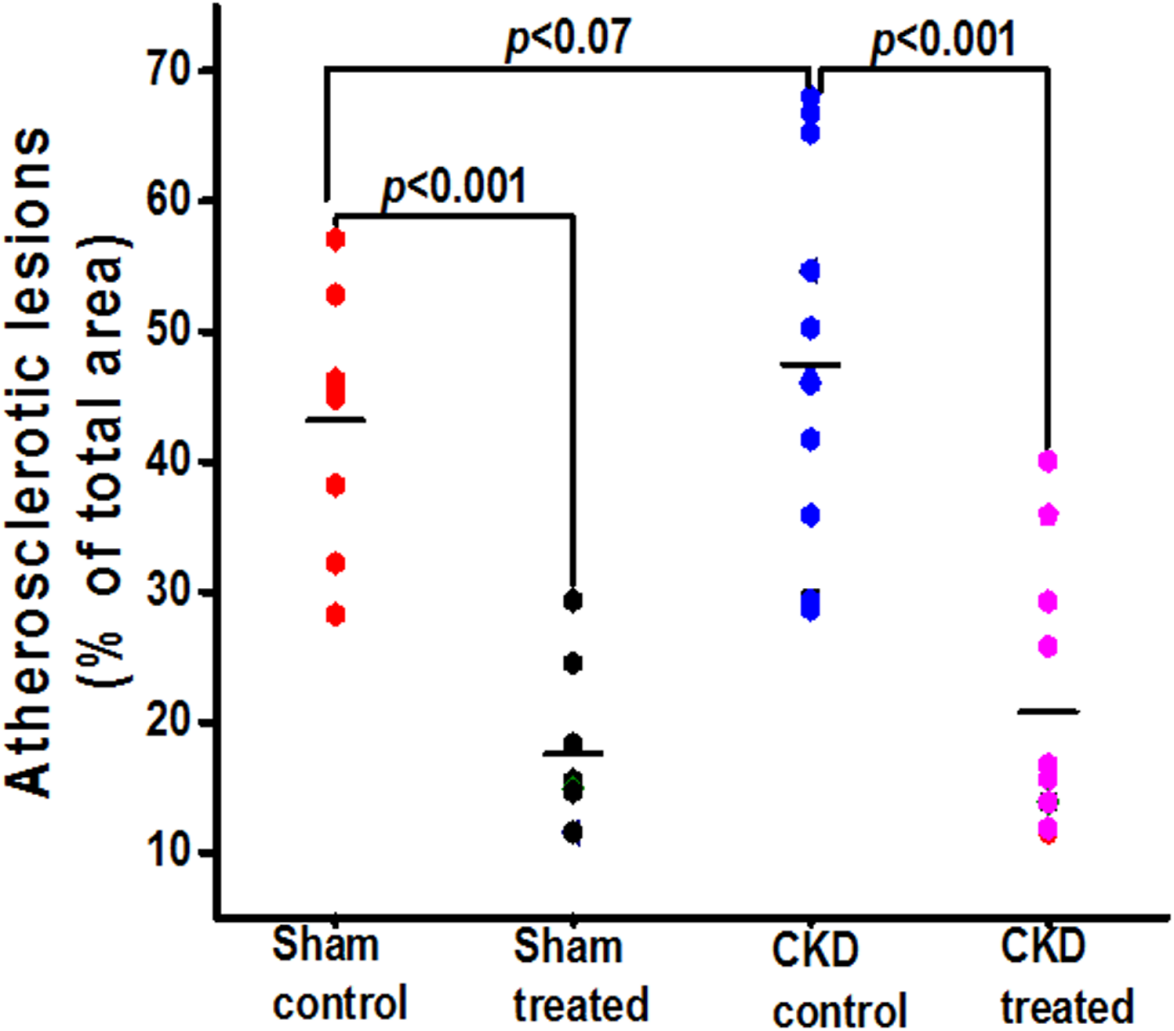
Total atherosclerosis area for the entire aortas in all groups of mice at the end of 16 weeks of therapy. Graph shows the quantification of total atherosclerotic lesion area on Sudan IV staining of entire separated aortas in all groups of mice. The black horizontal bar represents means.

**Fig 5:**
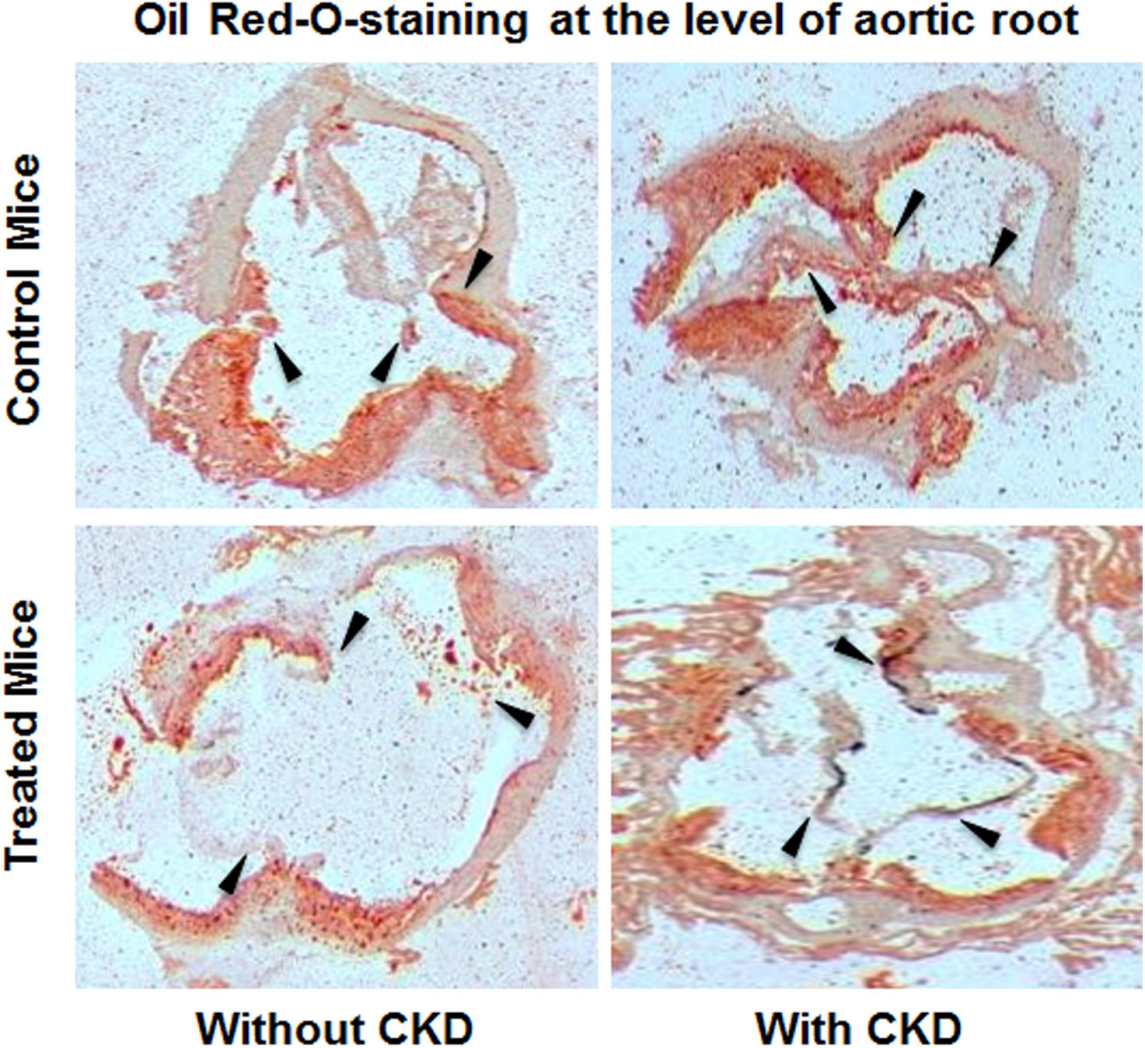
Isolated aortic bulb analysis with Oil Red O staining in CKD groups. Representative images of Oil Red O staining for lipid deposits in ascending aortas of all groups of mice. Red indicates the lipid-laden areas representing atherosclerotic lesion coverage. Black arrows represents the aortic root / valve leaflet in the section.

Figs 3–5 show the postmortem data for the extent and severity of atherosclerosis in both CKD and non-CKD groups of mice. HCQ significantly reduced the area of atherosclerosis as evidenced on *en face* Sudan IV staining of the whole aorta (Figs 3 and 4) in the CKD treatment group (20.75 ± 3.3%) compared to CKD control mice (47.48 ± 4.1%) (*P*<0.001). This was accompanied by a significant reduction in the severity of atherosclerosis in the aortic bulb on Oil Red O examination in CKD treatment mice compared to control CKD (Fig 5). Similar beneficial effects were confirmed in non-CKD/sham groups, with a reduction in atherosclerotic plaques from 43.18 ± 3.7% (untreated) to 17.53± 2.4% (in treated group (*P*<0.001), validating the results of a prior study in a non-CKD state (Figs 4 and 5)[16]. This was also confirmed in the two-way ANOVA model (β estimate ± SE: -23.5 ± 5.2, *P* < .0001).

### Impact of HCQ on vascular functions

**Fig 6:**
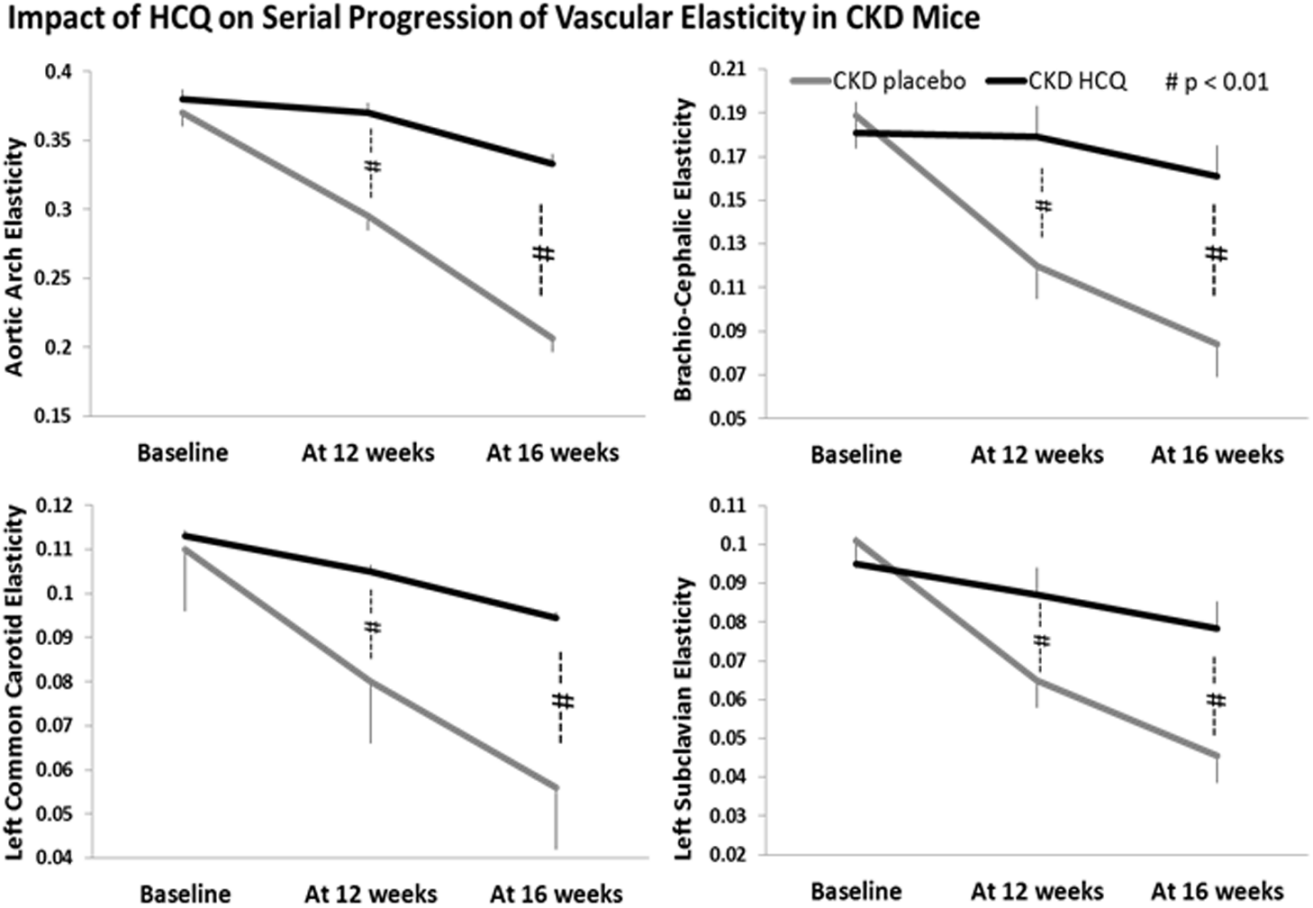
Quantitative analysis of vascular elasticity in CKD groups. Quantification of vascular wall elasticity as visualized by M-mode intravital ultrasound echography using VisualSonics Vevo 770 in abdominal aortas. The elasticity of the major vessels, *i*.*e*., the movement of vessel wall between the systole and diastole, as well as blood velocity within these vessels were significantly better in CKD mice treated with HCQ. *P < 0.05 compared with CKD placebo mice. AA, aortic arch; BCA, brachio-cephalic artery; LCCA, left common carotid artery; LSA, left subclavian artery.

**Fig 7:**
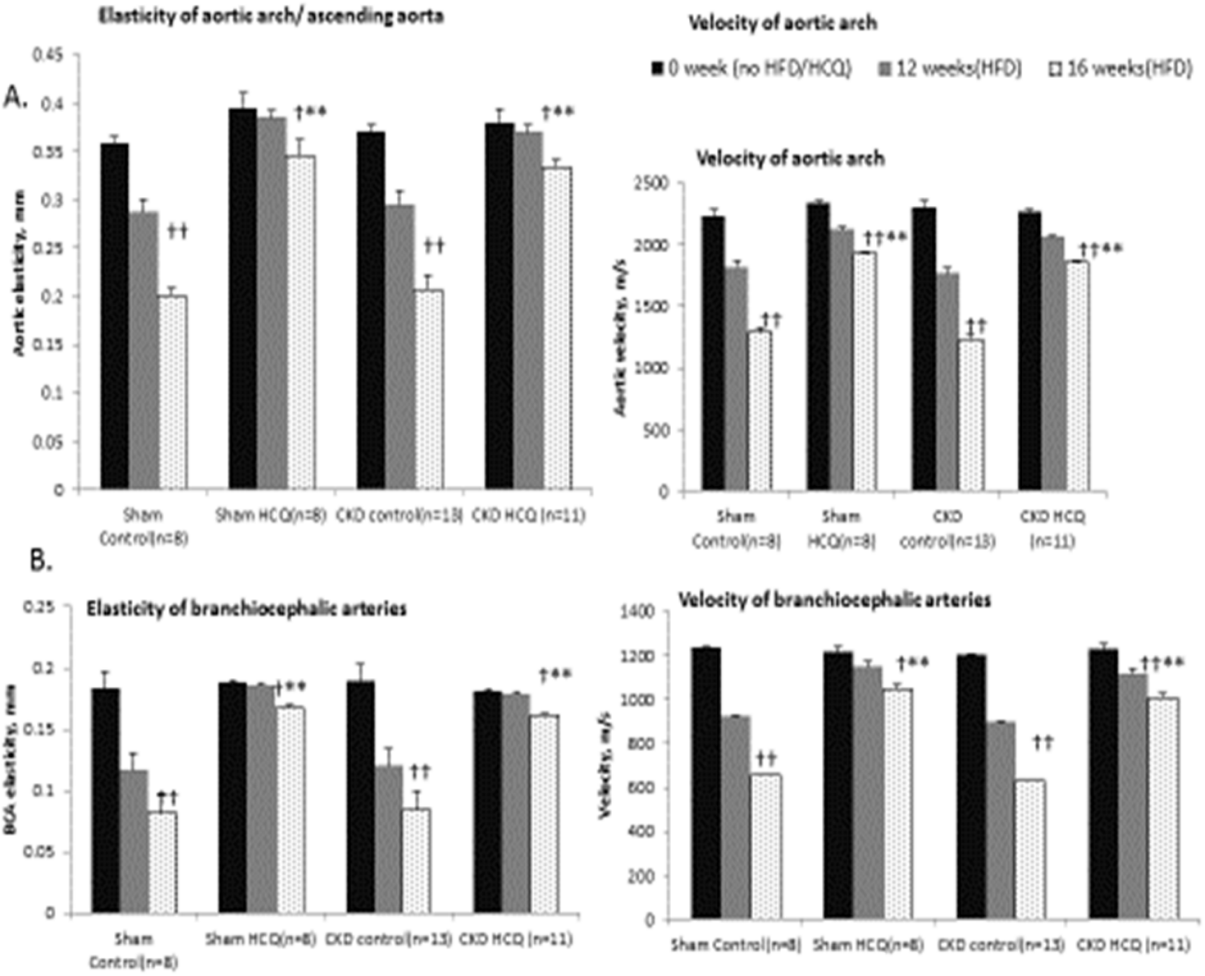
Progression of vascular elasticity and velocities in aortic arch and brachiocephalic arteries in all groups of mice. Quantitative analysis of serial changes in the elasticity and velocity of the aortas and brachiocephalic arteries in all groups of mice. ^†^p<0.05, ^††^p<0.01, as compared to the zero time of individual group, **p<0.05 as compared to the untreated control of each group.

We measured the vascular endothelial function through the antemortem measurements of vascular elasticity and blood-flow dynamics for all major vessels on AUE using the M mode and Doppler mode. We observed a significant and progressive reduction in vascular elasticity (Figs 6 and 7) and blood flow parameters (Fig 7) in all major vessels over the study period in all groups of mice, in tandem with development of atherosclerosis. Mice receiving HCQ therapy had better preservation of the functional indices of aortic and vascular compliance as determined through the M-mode and Doppler assessments (Fig 7) compared to the mice not receiving HCQ. In parallel with histologic protection, the beneficial effects for HCQ were seen equally potent in the presence of uremic milieu when compared to those with normal renal function (sham groups).

### Impact of HCQ on inflammation and select biochemical parameters

HCQ is an anti-inflammatory drug and inflammation has been proposed to have a significant role in many complications of CKD including CVD and renal failure progression. We examined the impact of HCQ on inflammation, renal functions, and lipids parameters.

We evaluated the impact of HCQ on systemic inflammation by measuring the serum levels of hsCRP. Therapy with HCQ did not affect the levels of hsCRP in mice with CKD (Table 2). To confirm these results, we conducted CRP measurements in another laboratory at our institution and obtained similar results (data not shown). Treatment with HCQ did not affect IL-6 levels or VCAM levels in the CKD group (Table 2). Similarly, there was no impact of HCQ on the lipid profile in these hyperlipemic mice or on the severity of renal failure between both CKD groups (Table 2).

**Table 2:** Serum biochemical parameters at the time of sacrifice.

	Total cholesterol (mg/dL)	LDL (mg/dL)	IL-6 pg/mL	sVCAM-1 ng/mL	CRP ng/mL
**Sham control (n = 8)**	883.2±60.9	532±34	494.8±138.8	714.3±50.1	14.6±9
**Sham treated (n = 8)**	842.9±41.9	526±25.9	470.2±164.6	679.8±42.8	13.6±17
**CKD control (n = 13)**	957.4±39.5	616.3±44.3	394.8±143.9	838.8±3	33.9±2*
**CKD treated (n = 11)**	1010.9±60.5	609.7±29.8	369±130.9	826.7±29.5^†^	29.9±1^†^

Serum biochemical parameters for total cholesterol, low-density lipoprotein cholesterol (LDL), interleukin-6 (IL-6), soluble vascular cell-adhesion molecule-1 (sVCAM-1) and high sensitivity C-reactive protein (CRP) at the study end, at the time of sacrifice in all groups of mice.

Data presented as means±SD

Kruskal-Wallis test followed by Dunn post-hoc test

*P<0.01 vs. sham control

†*P*<0.05 vs. sham treated.

## Discussion

Our study demonstrates that HCQ confers a significant functional and histological protection against vascular stiffness and atherosclerosis in presence of uremic milieu. HCQ substantially reduced the severity of atherosclerosis (by ~60%) in these atherogenic mice, improved vascular compliance, and prevented vessel wall thickness. More importantly, the anti-atherosclerotic and vasculoprotective effects of HCQ were not mitigated by and appeared to be equally potent in the presence of uremic milieu.

The principle outcome of our study is the validation of strong atheroprotective effects for HCQ even in the presence of CKD. HMG Co-A inhibitors (statins), the mainstay for management of atherosclerotic CVD, have strong evidence for their efficacy in animal atherosclerosis models and in human clinical trials in the presence of normal renal function[25–27]. However, when Ivanovski et al. evaluated their efficacy in a similar animal model in the presence of uremia, they found that statins do not reduce the severity of atherosclerosis in the uremic milieu[8]. Clinically as well, statins have repeatedly failed to reduce cardiovascular or all-cause mortality when studied for their efficacy in high CVD and CKD/ESKD populations[5, 6]. Together, these statin data suggest that the therapeutic efficacy of an anti-atherosclerotic agent proven in the presence of normal renal function does not automatically translate into an effective therapy in the presence of uremic milieu and that statins as a sole strategy for the management of CVD in CKD is inadequate. Our study provides evidence for an *in vivo* anti-atherosclerotic efficacy for HCQ in CKD. Previous studies demonstrating that HCQ is safe to use in the presence of advanced CKD thus make clinical trials feasible.

Clinically, endothelial dysfunction with resultant vascular stiffness, inflammation, and accelerated atherosclerosis are prominent factors for CVD associated with CKD[28]: Endothelial injury and dysfunction with reduction in NO availability, and resultant increase in vascular stiffness are some of the earliest events evident in CV injury. Endothelial integrity and NO availability have important roles in the maintenance of vascular tone, control of inflammation, smooth muscle cell proliferation and migration, and thrombogenesis, fibrinolysis,[29] and eventual downstream worsening of atherosclerosis.

Ghigo et al. through their *in vitro* studies have shown that HCQ stimulates vascular endothelial cell NO release[30]. Multiple *in vivo* studies in animal models of lupus, diabetes, and metabolic syndrome have since shown a beneficial impact of HCQ vascular endothelial cell function, as judged by improvement in endothelial dependent vasodilation[31–34]. Even studies involving human rheumatological cohorts have shown that long-term use of low-dose HCQ is associated with an improvement in indices of vascular compliance, e.g. aortic pulse wave velocity (APWV) and reduction in new-onset hypertension[14, 35, 36].

Endothelial dysfunction as measured by AUE echography has been strongly associated with the development and severity of atherosclerotic plaque in apoE-/- mice models[21, 24], and blinded AUE assessment of major vasculature has excellent validity with the severity of vascular disease and atherosclerosis. Our findings indicate that HCQ in low doses similar to those employed for chronic use in the human population has strong protective effects on the structural and functional aspects of vascular health as reflected by better preserved vascular wall thickness, and vascular flow indices and elasticity across multiple vascular beds.

In our study, HCQ failed to reduce the degree of inflammation as reflected by the serum levels of hsCRP among mice with or without CKD. Retrospective database analyses have suggested a possible beneficial role for HCQ on the lipid profile, with some suggesting an improvement in LDL and HDL values[37–39]. In our study, we did not demonstrate a benefit of HCQ on the conventional lipid profile. We also found no significant impact of HCQ on the degree of renal dysfunction among treated vs. control mice with CKD, indicating that the beneficial effect of HCQ was unrelated to improvement in renal function (Table 1). Thus, our results suggest that the beneficial actions of HCQ toward atherosclerosis occurs even in the absence of significant anti-inflammatory, renoprotective, or anti-lipidemic actions, and might be related to alternate mechanisms.

Analyses of various *in vitro*, *in vivo*, and human cohort data indicate that HCQ has many pleiotropic effects, e.g. reduction in oxidative stress, improvement in insulin sensitivity and metabolic syndrome[14–19, 22], improvement in vascular endothelial function with improved availability of eNOS activity, and inhibition of autophagy[40] with concerns for heightened maladaptive autophagy in endothelial and vascular smooth muscle cells[41–43] in tissues with accelerated atherosclerosis, etc. Razani et al. in their metabolic studies also found that chloroquine, a precursor molecule of HCQ, inhibits atherosclerosis through a p53-dependent stress pathway[17]. More importantly, the reasons for worsening CVD and its refractoriness towards conventional therapies in the presence of uremic milieu are multifactorial and at present unknown. Thus, additional studies are needed to further define the factors associated with the worsening CVD with uremia and the potential mechanisms by which HCQ affords its beneficial effects on atherosclerosis in uremic milieu.

Although our study does not provide mechanistic insight, we believe it is clinically relevant because it proposes a therapy for CVD with a drug that has been in use for several decades, even in patients with advanced CKD and ESKD[44, 45]. This suggests a potential for evaluation of HCQ for cardiovascular events in CKD patients. Further studies are needed to clarify the mechanisms through which HCQ confers its benefits.
